# Asymmetric and Distant Effects of a Unilateral Lesion of the Primary Motor Cortex on the Bilateral Supplementary Motor Areas in Adult Macaque Monkeys

**DOI:** 10.1523/JNEUROSCI.0904-18.2018

**Published:** 2018-12-12

**Authors:** A. Contestabile, R. Colangiulo, M. Lucchini, A.-D. Gindrat, A. Hamadjida, M. Kaeser, J. Savidan, A.F. Wyss, E.M. Rouiller, E. Schmidlin

**Affiliations:** Department of Neurosciences and Movement Sciences, Section of Medicine, Faculty of Sciences and Medicine, Fribourg Center of Cognition, University of Fribourg, Chemin du Musée 5, CH-1700 Fribourg, Switzerland

**Keywords:** cortical lesion, diaschisis, manual dexterity, motor cortex, nonhuman primate

## Abstract

A restricted lesion of the hand area in the primary motor cortex (M1) leads to a deficit of contralesional manual dexterity, followed by an incomplete functional recovery, accompanied by plastic changes in M1 itself and in other cortical areas on both hemispheres. Using the marker SMI-32 specific to pyramidal neurons in cortical layers III and V, we investigated the impact of a focal unilateral M1 lesion (hand representation) on the rostral part (F6) and caudal part (F3) of the supplementary motor area (SMA) in both hemispheres in nine adult macaque monkeys compared with four intact control monkeys. The M1 lesion induced a consistent interhemispheric asymmetry in density of SMI-32-positive neurons in F3 layer V (statistically significant in 8 of 9 lesioned monkeys), highly correlated with the lesion volume and with the duration of functional recovery, but not with the extent of functional recovery itself. Such interhemispheric asymmetry was neither present in the intact monkeys, as expected, nor in F6 in all monkeys. In addition, the M1 lesion also impacted on the basal dendritic arborization of F3 layer V neurons. Neuronal density was clearly less affected by the M1 lesion in F3 layer III compared with layer V. We interpret the remote effect of M1 lesion onto the density of SMI-32-positive neurons and dendritic arborization in the SMAs bilaterally as the consequence of multiple factors, such as changes of connectivity, diaschisis and various mechanisms involved in cortical plasticity underlying the functional recovery from the M1 lesion.

**SIGNIFICANCE STATEMENT** The motor system of macaque monkeys, in addition to be similarly organized as in humans, is a good candidate to study the impact of a focal lesion of the main contributor to voluntary movements, the primary motor cortex (M1), on non-primary motor cortical areas also involved in manual dexterity, both at behavioral and structural levels. Our results show that a unilateral permanent lesion of M1 hand area in nine monkeys affects the interhemispheric balance of the number of SMI-32-positive pyramidal neurons in the cortical layer V of the supplementary motor area, in a way strongly correlated to the lesion volume and duration of the incomplete functional recovery.

## Introduction

In primates, the hand area of the primary motor cortex (M1 or F1) and multiple functionally distinct premotor areas contribute to complex motor behaviors, such as manual dexterity, via corticocortical interactions. Among them, two specific premotor areas play a particular role: first, the ventral premotor cortex (PMv-r or F5) is involved in visually guided movements (Shimazu et al., 2004) and is functionally connected to M1 (Schmidlin et al., 2008); second, the supplementary motor area (SMA-proper or F3) is involved in the sequential control of movements, and also directly controls distal movements of the hand (Maier et al., 2002; Boudrias et al., 2010). Structural changes at distant, but directly connected regions have been shown in New World monkeys subjected to unilateral lesion of M1, in the form of a rewiring of the remaining F5-to-M1 projection redirected toward the primary somatosensory cortex (Dancause et al., 2005).

**Figure 1.**
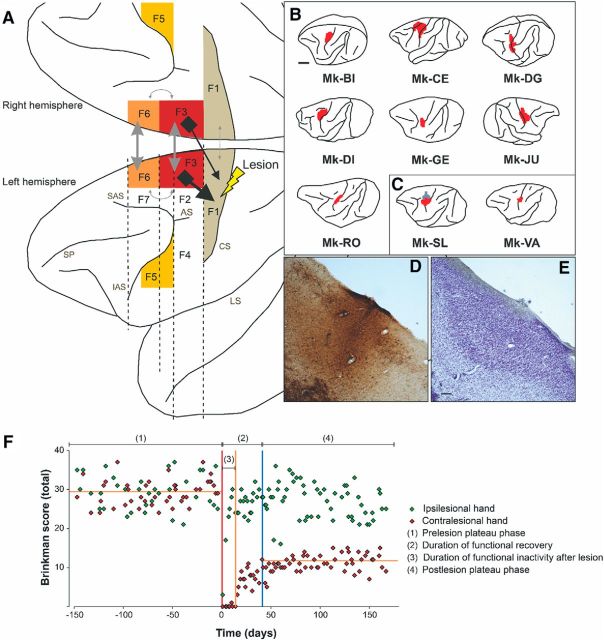
***A***, Schematic representation of a macaque brain showing the location of pre-SMA (area F6, orange), SMA-proper (area F3, red), and their anatomical connectivity with the M1 (F1, beige). F2 and F7 correspond to the caudal and rostral parts of the dorsal premotor cortex (PMd), respectively. F4 and F5 correspond to the caudal and rostral parts of the PMv, respectively. The straight double-head arrows in gray represent the interhemispheric connections, with the notion that interhemispheric callosal connections are less dense in F1 than in F3 and F6. Furthermore, F3 projects more strongly on the ipsilateral F1 than on the contralateral F1 (black arrows). The curved double head gray arrows represent the interconnections between F3 and F6. Importantly, F6 does not project to F1 and reciprocally. As explained in the method section, the limit between F3 and F6 was displaced 3 mm rostral to the genu of the arcuate sulcus, to ensure full inclusion of F3 in the histological analysis. ***B***, ***C***, Schematic representation of the extent and location of the F1 lesion (red area) in the nine monkeys involved in the present study, as seen in transparency on the cortical surface. The two monkeys in ***C*** were treated with anti-Nogo-A antibody after the lesion, whereas the seven other monkeys (***B***) were untreated. ***D***, ***E***, Photomicrographs of coronal histological sections through F1 (Mk-DI) showing the induced permanent lesion in the hand representation. Scale bar, 100 μm. The adjacent sections derived from two series were processed to visualize SMI-32 staining (***D***) or Nissl staining (***E***). ***F***, Graphical representation of typical behavioral performance of macaque monkeys in the modified Brinkman board task. The manual performance of each hand is given by the score (number of pellets retrieved in the first 30 s of the task from the randomly distributed wells) as a function of time (days) before and after a lesion of the hand representation in F1. Day 0 corresponds to the day of the lesion (vertical red line). Relevant for the present report are the scores (for the contralesional hand) pre-lesion [(1) the horizontal orange line is the median score] and postlesion [(4), the horizontal orange line is the median score]. The total duration of functional recovery of manual dexterity (2) after lesion is given by the time interval between the lesion (red vertical line) and the onset of the postlesion plateau (vertical blue line). The duration of total loss of manual dexterity [(3) score = 0] is the time interval between the lesion and the first consistent successful attempt to retrieve a pellet (vertical orange line). SAS, Superior arcuate sulcus; IAS, inferior arcuate sulcus; AS, arcuate spur; SP, sulcus principalis; LS, lateral sulcus; CS, central sulcus.

The rostral part of SMA (pre-SMA or F6) differs functionally from its caudal part (F3; Wiesendanger, 1986; Matsuzaka et al., 1992; Luppino et al., 1993; Dum and Strick, 1996; Liu et al., 2002). F3 is reciprocally connected with M1, both regions being at the origin of corticospinal (CS) projections, which is not the case of F6 (Dum and Strick, 1991; Luppino et al., 1993, 1994). Transcallosal interconnections exist between F3 and the hand representation of M1 although these projections are less dense than the F3–M1 interconnections within the same hemisphere (Rouiller et al., 1994; Fig. 1*A*).

In case of unilateral lesion of M1, there is evidence that the ipsilesional SMA may contribute to functional recovery (McNeal et al., 2010; Morecraft et al., 2015). Following a lesion affecting M1, the density of the projection from M1 to SMA will decrease, and thus the influence of M1 onto SMA will decline. Similarly, because of a decrease of target neurons in M1, the projection from SMA to M1 may also be impacted retrogradely. This is reminiscent of the decrease of neuronal sensitivity to SMI-32 antibody and shrinkage of the soma, which has been observed in corticospinal neurons in M1 following cervical cord lesion (Wannier et al., 2005; Beaud et al., 2008).

The corticocortical connections involve mostly pyramidal neurons of origin in layers II, III, and V, selectively stained by SMI-32 (Liu et al., 2002; Wannier et al., 2005; Beaud et al., 2008; García-Cabezas and Barbas, 2014). This allows the comparison of pyramidal neuronal labeling in the lesioned hemisphere and in the intact hemisphere. Here, we focused on assessing histological and cellular changes occurring bilaterally in F3 and F6 (as possible control region) after a permanent and unilateral lesion of the hand representation in M1 in macaque monkeys. Assessing the structural changes in SMA induced by the lesion of a distant, but densely interconnected cortical area (M1), and their possible link to functional recovery, may contribute to better identify the morphological support of cortical plasticity following a cortical lesion in human, e.g., corticocortical changes following a motor stroke (Ward and Cohen, 2004; Bestmann et al., 2010; Schaechter et al., 2012).

The aim of the present study was to test the following hypotheses: (1) A unilateral lesion of M1 impacts at distance onto SMA but asymmetrically in each hemisphere, producing an interhemispheric asymmetry of pyramidal neuronal density in SMA (Figs. 1*A*, Fig. 5*B*). (2) The impact of the M1 lesion on SMA in each hemisphere depends on the size of the M1 lesion. (3) As SMA is involved in the functional recovery, the impact of the M1 lesion on SMA is also correlated with the extent and/or duration of functional recovery. (4) The impact of the unilateral M1 lesion is different onto F3 (SMA-proper) and F6 (pre-SMA).

To test these hypotheses, we compared the density and morphology of pyramidal neurons using SMI-32 staining in F3 and F6 across both hemispheres in four intact macaque monkeys and in nine macaque monkeys subjected to unilateral lesion of the hand area in M1.

## Materials and Methods

### 

#### 

##### Macaque monkeys.

The histological analysis was conducted on 13 adult macaque monkeys (*Macaca fascicularis* and *M. mulatta*; Table 1). All procedures were conducted in accordance with guidelines of the federal and local veterinary authorities (veterinary authorizations FR 24/95/1; FR 44/92/3; FR 157/01; FR 157/03; FR 157/04; FR 156/04; FR 156/06; FR 157e/06; FR 185/08: FR 17/09; FR 192/07E; FR 192/07). Several monkeys were already involved in previous reports addressing distinct issues related to motor cortex lesion (Liu and Rouiller, 1999; Kaeser et al., 2010, 2011; Schmidlin et al., 2011; Bashir et al., 2012; Hamadjida et al., 2012; Hoogewoud et al., 2013; Wyss et al., 2013; Savidan et al., 2017). Nine animals (Mk-IC, Mk-IE, MK-IR, Mk-IZ, Mk-BI, Mk-CE, Mk-DG, Mk-DI, Mk-GE, Mk-JU, Mk-RO, and Mk-SL) were subjected to a unilateral lesion of the hand region in M1 by using intracortical microinjections of ibotenic acid, as previously reported (Liu and Rouiller, 1999; Kaeser et al., 2010; Hamadjida et al., 2012; Wyss et al., 2013; Murata et al., 2015; Savidan et al., 2017). We functionally identified the M1 hand representation using intracortical microstimulation in all animals (except Mk-DI) with standard stimulation parameters as reported in detail previously (Wyss et al., 2013, Savidan et al., 2017). As stated by Savidan et al. (2017), Mk-DG was subjected to a first unilateral and permanent cortical primary lesion of M1, which predominant long-term effects are analyzed in our study, whereas short-term effects of a secondary M1 lesion in the opposite hemisphere were most likely modest as the euthanasia of this animal was performed shortly after the secondary lesion. The other four monkeys (Mk-IC, Mk-IE, Mk-IR, and Mk-IZ) had no M1 lesion (intact monkeys used as controls). Two of the nine lesioned monkeys were treated with anti-Nogo-A antibody (Mk-SL and Mk-VA; Kaeser et al., 2010; Hamadjida et al., 2012; Wyss et al., 2013). Comprehensive descriptions of the anti-Nogo-A antibody treatment procedure were published earlier (Freund et al., 2007, 2009; Hamadjida et al., 2012). In short, a small craniotomy was made in the occipital bone to position a subdural cannula (Alzet osmotic pump) delivering the antibody during 4 weeks. At the end of the behavioral assessments, the animals were euthanized under deep anesthesia obtained with an intraperitoneal overdose of pentobarbital sodium (90 mg/kg body weight), as previously reported (Kaeser et al., 2010; Wyss et al., 2013).

**Table 1.** Summary of the properties of each monkey included in the study

Monkey groups	Intact				M1 lesioned								
					Untreated							Treated with anti-Nogo-A antibody	
Monkey IDs	Mk-IC	Mk-IE	Mk-IR	Mk-IZ	Mk-BI	Mk-CE	Mk-DG	Mk-DI	Mk-GE	Mk-JU	Mk-RO	Mk-SL	Mk-VA
Species	fasc	mul	fasc	fasc	fasc	fasc	fasc	fasc	fasc	fasc	fasc	fasc	fasc
Gender	M	M	F	M	M	M	M	F	F	M	M	M	M
Side of M1 hand lesion	—	—	—	—	L	L	L (R)*^c^*	L	L	R	L	L	L
Total volume of the M1 lesion in gray matter, mm^3^	0	0	0	0	20.1	112.8	32.2	68.5	48.7	63	14	78.2	20
Degree of functional recovery from M1 lesion; total score, %*^a^*	—	—	—	—	74	42	58	39	38	39	98	73	87
Degree of functional recovery, V slots	—	—	—	—	94	59	69	71	57	46	100	77	87
Degree of functional recovery, H slots	—	—	—	—	36	9	45	7	11	29	90	77	91
Age at time of lesion (sacrifice for control monkeys), rounded 0.5 year	10	5.5	6	8	5	4.5	10	10	5	5	4	5.5	5.5
Time interval lesion; sacrifice, d	—	—	—	—	265	385	212	187	210	310	222	265	366
Weight at time of lesion, kg	—	—	—	—	5	3.8	8.6	3.8	2.8	3.6	3.2	4.6	4.9
Volume ibotenic acid, μl	—	—	—	—	29.7	40	24	39.7	13	40	18	18	15.5
Duration of recovery, d*^b^*	—	—	—	—	35	113	30	42	8	70	30	58	20(120)

M, Male; F, female; R, right; L, left; fasc, *M. fascicularis*; mul, *M. mulatta*.

*^a^*Expressed in percentages of postlesion total score at plateau divided by pre-lesion total score in the modified Brinkman board task: all slots. The next two lines are the degrees of functional recovery given separately for the vertical (V) and horizontal (H) slots. The next two lines are the degrees of functional recovery given separately for the V and H slots.

*^b^*Time interval (Fig. 1*F*) from the day of lesion to the beginning of postlesion plateau, as defined by Kaeser et al. (2011). For Mk-VA, there was a second plateau of recovery that occurred 120 d postlesion, possibly linked to the anti-Nogo-A antibody treatment in this animal (Wyss et al., 2013).

*^c^*158 d after the permanent lesion with infusion of ibotenic acid, corresponding to 58 d before euthanasia.

##### Histology and neuroanatomical reconstruction.

**Figure 2.**
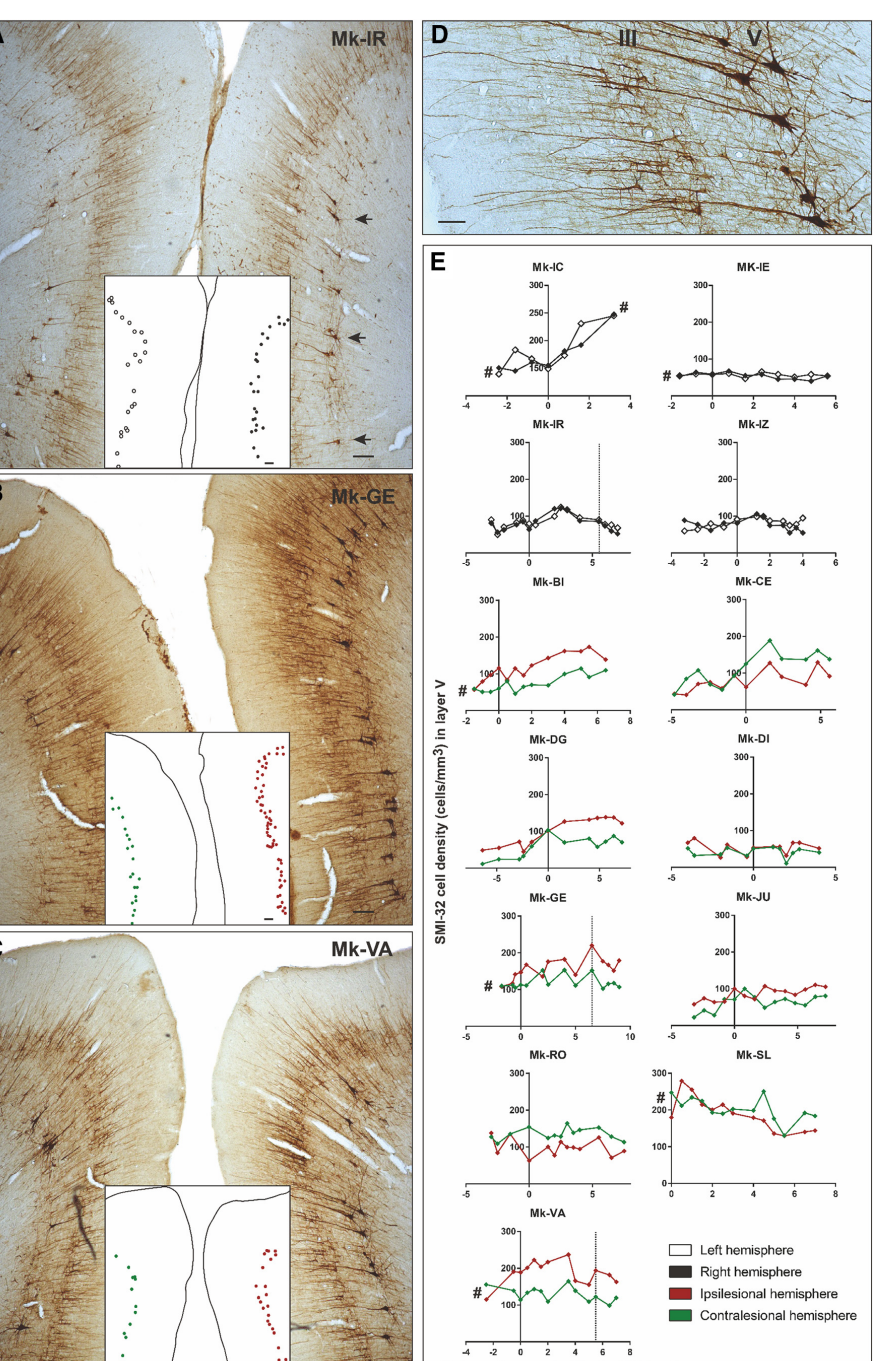
***A***–***C***, Photomicrographs of coronal brain histological sections of an intact macaque monkey (***A***; Mk-IR), a lesioned monkey (***B***; Mk-GE) and a lesioned monkey treated with the anti-Nogo-A antibody (***C***; Mk-VA), all stained with SMI-32. Scale bars, 100 μm. The medial part of SMA of each hemisphere is visible in these coronal sections. The localization of the SMI-32-positive neurons taken into account for the Sholl analysis is shown in ***A*** (arrows). A dot representation of layer V SMI-32-positive neurons included in our analyses is illustrated in the white insets. The layer V SMI-32-positive neurons in the lesioned hemisphere (images, right) are indicated with red dots, and the ones in the intact hemisphere (image, left) are indicated with green dots in ***B***–***C***. Higher-magnification photomicrograph of a coronal section of F3 in the right hemisphere of a macaque monkey (Mk-VA). Scale bar, 40 μm. The layers III and V are visible with the corresponding SMI-32-positive pyramidal cells and their identifiable dendritic arborization. ***E***, Graphs representing the rostrocaudal gradient (from F6 to F3) of SMI-32-positive cell density in layer V of all monkeys. The cell density for each hemisphere is plotted as a function of the distance from the F3–F6 border, which has been set to 3 mm rostrally to the genu of the arcuate sulcus. Negative distance values belong to F6 and positive distance values belong to F3. Vertical dashed lines (Mk-IR, Mk-GE, and Mk-VA) correspond to quantification of SMI-32-positive neurons observed in the photomicrographs of ***A***–***C***, respectively. The # symbol was used to indicate that the analyzed cortex region was not complete (sections lacking for the analysis).

After euthanasia, the brain was cut in the frontal plane into 50-μm-thick frozen sections with both hemispheres facing each other on the same slide (Fig. 2*A*). Sections were collected in 5 or 8 consecutive series. One series was Nissl stained and another consecutive series was immunoreacted for the marker SMI-32 (Sternberger and Sternberger, 1983), as previously reported (Wannier et al., 2005; Beaud et al., 2008, 2012). Histological structures of interest were vectorised using Neurolucida 9 (MBF Bioscience) with a computer-interfaced Olympus BX40 microscope (Olympus Schweiz AG), a computer-controlled motorized stage (Märzhäuser Wetzlar GmbH, type EK 32 75 × 50) and a digital camera (Olympus U-PMTVC). Frontal brain sections were examined under bright-field illumination. The Photomicrographs taken with the digital camera were further edited in CorelDraw (color, brightness, and contrast were not modified). The quantification of the structures of interest was then performed with the software Neuroexplorer (MBF Bioscience) comparing the two hemispheres on the same section. At that step, the investigator was blinded against information on animal group (control vs lesion) and side of the cortical lesion.

The extent of the M1 cortical lesion on each section was defined as the cortical zone showing a loss of SMI-32-positive neurons in layer V of M1, then perpendicularly extended up to the cortical surface and down to the border between white matter and layer VI. Finally, the volume of the M1 cortical lesion (Fig. 1*B–E*; Table 1) was assessed by integrating the consecutive sections, using the Cavalieri's method, as previously described (Pizzimenti et al., 2007; Wyss et al., 2013). SMA (F3 and F6) in both hemispheres was delineated from M1 or PM laterally and from cingulate motor area (CMA) ventrally, based on cytoarchitectural landmarks (Liu et al., 2002). The number of SMI-32-positive neurons in layers III and V was established on each SMI-32 stained section and the surface of the delineated F3 or F6 was calculated in relation with the defined limits. The cellular density of F3, respectively F6, was defined on individual sections as the number of identified SMI-32-positive cells in that area in one hemisphere divided by the corresponding volume of SMA (given by the thickness of the section multiplied by the area-of-interest). The cell density was assessed at regular intervals along the rostrocaudal axis (Fig. 2*E*). The criteria used to include a neuron were the following: (1) the neuron is SMI-32-positive; (2) the soma, the nucleus or the nucleolus and at least four proximal dendrites are identified; and (3) the neuron is located in the cortical layer III or in layer V. The analysis on the light microscope was conducted at a magnification of 100×. In addition, to test the liability of the neuronal identification method, cell counts were repeated at a magnification of 200× in several sections from different monkeys, yielding identical results as those obtained at 100× magnification. In each analyzed histological section, all SMI-32-labeled neurons within the delineated area were counted, corresponding to exhaustive sampling method instead of stereology (Fregosi and Rouiller, 2017). All histological samples across monkeys and hemispheres were analyzed according to the same procedure.

Along the rostrocaudal axis, the limit between F3 and F6 was estimated at the level of the genu of the arcuate sulcus (Liu et al., 2002), even though this boundary is difficult to establish, especially on frontal sections. Moreover, considering functional properties, the transition from F3 to F6 is progressive, without abrupt limit (as discussed in detail by Liu et al., 2002). Therefore, histological sections located up to 3 mm rostral to the genu of the arcuate sulcus were included in the F3 block to ensure that F3 was entirely considered. Further rostrally located sections then were included in the F6 block. Along the mediolateral axis, the analysis of each histological section was limited to the territory of SMA (F3 and F6) located in the vertically oriented portion of the medial wall of the hemisphere (Fig. 2*A–C*), whereas the zone of F3 or F6 including the curvature on the top of the medial wall and the more lateral portion of cortex on the surface of the hemisphere (Liu et al., 2002) were excluded. Indeed, this curved cortical zone exhibits a more variable layer arrangement and neuronal orientation than the medial wall, preventing a perfectly mirror-like analysis of comparable zones between both hemispheres. Similarly, the ventral part of SMA in the curvature of the cingulate sulcus toward the CMA limit was also excluded from the analysis (Fig. 2*A–C*).

##### Interhemispheric difference in cell density.

**Figure 3.**
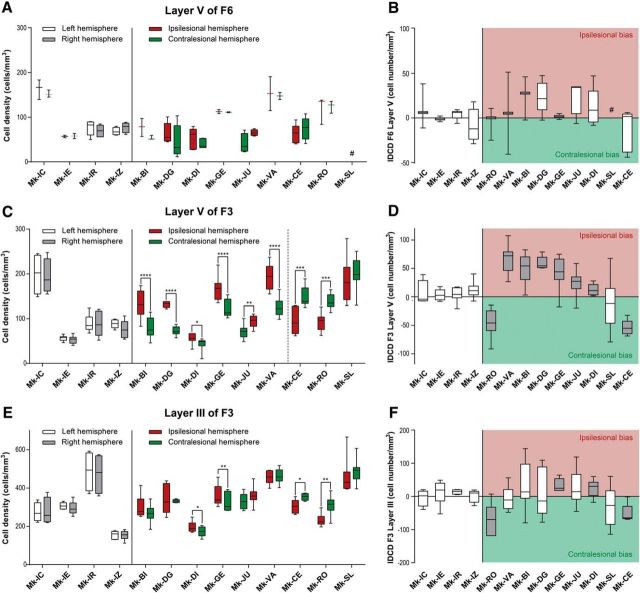
Box plots of interhemispheric morphological data obtained in the three investigated cortical regions (layer V of F6; layer V of F3; layer III of F3): cell density on both hemispheres in ***A***, ***C***, and ***E***, and IDCD in corresponding regions in ***B***, ***D***, and ***F***. ***A***, ***C***, ***E***, Box plots showing the SMI-32-positive cell densities in layer V of F6 (***A***), as well as in layer V (***C***) and layer III (***E***) of F3 in each hemisphere for each monkey. In the four intact monkeys, the cell density in the left and right hemispheres is represented in white and gray, respectively. In the nine monkeys subjected to unilateral M1 lesion, the cell density in the ipsilesional and contralesional hemispheres is represented in red and green, respectively. As statistical test, a paired *t* test or Wilcoxon test was performed (*p ≤ 0.05, **p ≤ 0.01, ***p ≤ 0.001, ****p ≤ 0.0001), comparing the density in the two hemispheres in each consecutive histological section. The absence of asterisks means “not statistically significant” (*p* > 0.05). ***B***, ***D***, and ***F***, Box plots showing the IDCD of SMI-32-positive cells in layer V of F6 (***B***), as well as in layer V (***D***) and layer III (***F***) of F3 in each monkey. Lesioned animals, on the right, were ordered from left to right according to an increasing M1 lesion volume. The white boxes point to the animals with a non-statistically significant IDCD, whereas the gray boxes show the animals with a significant IDCD. ***B***, All boxes should appear in white as none of the IDCDs are statistically significant (a few boxes are too small to appear white at that scale). A positive IDCD corresponds to an ipsilesional bias in pyramidal SMI-32-positive neurons density, whereas a negative IDCD corresponds to a contralesional bias. In all plots, the ID of each individual monkey is indicated along the abscissa. #, missing data.

In each section, an interhemispheric difference in cellular density (IDCD) was obtained by subtracting the SMI-32-positive neuron density in the contralesional SMA (separately for F3 and F6) from the SMI-32-positive neuron density in the ipsilesional SMA (Fig. 3*B*,*D*,*F*). Positive IDCD means that more SMI-32 neurons were found in the ipsilesional SMA whereas negative IDCD implies a larger number of SMI-32 neurons in the contralesional SMA. In intact monkeys, IDCD was expected to be close to zero, whereas in monkeys subjected to M1 lesion, IDCD was hypothesized to be biased toward the negative side (see Fig. 5*B*, dashed line). The comparison between the two hemispheres was performed on each individual section, confronting the left SMA with the right SMA, as done for M1 in a previous report (Wannier et al., 2005). This way, the mirrored SMA territories analyzed on each side were comparable, both in terms of area and position.

##### Single-neuron reconstruction.

The reconstruction of selected SMI-32 labeled neurons was performed using the vectorization tool from the Neurolucida software. Using a 400× magnification, three SMI-32-positive pyramidal neurons of layer V per hemisphere per section were analyzed in four different sections in each of six representative monkeys: two intact monkeys and four monkeys with a M1 lesion. In the lesion subgroup, two monkeys had a large positive IDCD reflecting a bias toward the ipsilesional hemisphere (Mk-VA and Mk-GE), one monkey presented a balanced IDCD (Mk-JU) and one monkey exhibited an IDCD biased toward the contralesional hemisphere (Mk-CE). The following criteria were applied to specifically select SMI-32-positive neurons: (1) to be representative of the entire region-of-interest, one neuron was picked in each of the dorsal, middle, and ventral parts of the medial wall in F3 (Fig. 2*A*, arrows); and (2) at least intact primary and secondary dendrites and an apical dendrite reaching the layer III without interruption had to be clearly identified (Fig. 2*D*). For each reconstructed neuron, we divided the surface of the basal dendrites by the surface of the apical dendrites. To quantify the dendritic arborization, a Sholl analysis was performed (Wellman et al., 2007), by defining concentric virtual circles centered on the soma of the neuron of interest and counting the number of “dendritic intersections” between the described virtual circles located at regular distances from the soma (10 μm steps) and the identified dendritic arborization. We also determined the surface of the soma and the surface of the apical dendrite for each reconstructed neuron. A second level of quantification of dendritic arborization consisted in integrating the area under the curve of the ratio between dendritic intersections and distance from the soma to allow statistical comparisons and regression analysis (see Fig. 5*C*) as general assessment of the dendritic tree. It was not possible to apply this high-magnification dendritic analysis (Sholl analysis) to all selected monkeys because of suboptimal staining quality of some brain sections.

##### Behavior.

The manual dexterity of the nine monkeys subjected to unilateral lesion of M1 was tested using the “modified Brinkman board” task, challenging the opposition of the index finger and the thumb (precision grip) to retrieve small food pellets from 25 vertically oriented wells and 25 horizontally oriented wells randomly distributed over a Perspex board (Schmidlin et al., 2011). The effects of such M1 hand area lesion on this task have been reported in detail earlier (Liu and Rouiller, 1999; Kaeser et al., 2010; Bashir et al., 2012; Hamadjida et al., 2012; Hoogewoud et al., 2013; Wyss et al., 2013; Savidan et al., 2017). After M1 injury, the contralesional manual dexterity was dramatically affected, and the monkeys exhibited incomplete functional recovery, reaching a plateau of motor performance several months after the M1 lesion (Wyss et al., 2013; Fig. 1*F*). We focused here on the percentage of functional recovery for the contralesional hand following the unilateral lesion of M1 hand area, given by the median total score (number of pellets retrieved from both vertical and horizontal wells in 30 s) at postlesion plateau divided by the median total score at prelesion plateau × 100 (Schmidlin et al., 2011; Wyss et al., 2013). An example of prelesion and postlesion median total scores is illustrated in Figure 1*F* (2 horizontal lines). Similarly, the percentages of functional recovery were calculated separately for the vertical wells and the horizontal wells (Table 1). Moreover, the plot of scores was used to define the duration of total loss of manual dexterity and the duration of (incomplete) functional recovery until reaching a postlesion plateau (Fig. 1*F*).

##### Statistics.

To statistically assess the significance of asymmetric densities of SMI-32-positive neurons between the ipsilesional and contralesional hemispheres, we applied a paired *t* test or a Wilcoxon test (according to the data distribution) as the neuronal density was directly compared between both hemispheres on the same section, and so on for each individual animal. In a second level of statistical analysis, we compared the obtained IDCDs between individual animals using a Kruskal–Wallis test (Table 2) including Bonferroni's corrections (*p < 0.05, **p < 0.01, ***p < 0.001, ****p < 0.0001). The χ^2^ test was used to statistically evaluate and compare across subgroups of monkeys the frequency of occurrence of significant IDCDs (see Results). To assess the precise role played by the two structural factors, namely the lesion volume in M1 and the IDCD, on the functional recovery, we performed a linear model test on those three factors using the MATLAB R2017b function “fitlm”.

**Table 2.** *P* values of the pairwise *post hoc* analysis comparison of interindividual IDCDs across the M1 lesioned monkeys (*p* value with Bonferroni correction) using the positive Kruskal–Wallis test

	Mk-BI	Mk-DG	Mk-GE	Mk-JU	Mk-DI	Mk-SL	Mk-CE
Layer F3-V							
Mk-VA	1.0000	1.0000	1.0000	**0.0071**	**0.0035**	**0.0003**	**0.0045**
Mk-BI		1.0000	1.0000	0.6505	0.0864	**0.0068**	**0.0070**
Mk-DG			1.0000	0.0839	**0.0326**	**0.0392**	0.0606
Mk-GE				0.8389	**0.0500**	**0.0073**	**0.0030**
Mk-JU					1.0000	0.2247	**0.0070**
Mk-DI						1.0000	**0.0326**
Mk-SL							1.0000
Layer F3-II							
Mk-VA	1.0000	1.0000	0.6410	1.0000	1.0000	1.0000	0.8110
Mk-BI		1.0000	1.0000	1.0000	1.0000	0.8390	0.7830
Mk-DG			1.0000	1.0000	1.0000	1.0000	1.0000
Mk-GE				1.0000	1.0000	0.3630	0.0710
Mk-JU					1.0000	0.6980	0.1310
Mk-DI						0.8800	0.0870
Mk-SL							1.0000

The outlier Mk-RO (see text) is not included in this analysis. Statistically significant *p* values (*p* ≤ 0.05) are in bold type.

## Results

Immediately after ibotenic acid micro-infusion in M1 hand area, all animals presented a complete and flaccid paresis of the contralesional hand and were totally unable to perform the behavioral task during several days, corresponding to the duration of functional inactivity after lesion, as illustrated in Figure 1*F*. It was then followed by a progressive functional recovery until reaching a postlesion plateau.

### Neuronal density

We counted the number of SMI-32-positive neurons in the delimited F3 and F6 areas in both hemispheres (Fig. 2*A–D*) along the rostrocaudal axis in coronal sections from F6 to F3 in 13 macaque monkeys (Fig. 2*E*). The pyramidal SMI-32-positive neurons were then pooled per hemisphere and per animal. In F3 layer V, the range of SMI-32-positive neurons' densities in each hemisphere was comparable in the control (intact) monkeys and in the lesioned monkeys, varying across histological sections approximately from 15 cells/mm^3^ (Mk-DI) to 250 cells/mm^3^ (Mk-IC and Mk-SL; Fig. 3*C*). In F3 layer III, the cell density range varied between 100 cells/mm^3^ (Mk-IZ and Mk-DI) and 600 cells/mm^3^ (Mk-SL) both in intact and lesioned monkeys (Fig. 3*E*). This comparison suggests that after M1 lesion there was no substantial loss of SMI-32-positive neurons in both layers III and V of F3 and in layer V of F6 (Fig. 3*A*,*C*,*E*). Moreover, in the majority of monkeys, there were more SMI-32-positive neurons in F3 than more rostrally in F6 (Fig. 2*E*).

The assessment of cellular density in layer V of F6 showed no significant IDCD (Fig. 3*A*,*B*), both in intact animals and in unilaterally M1 lesioned monkeys. This observation indicates that SMI-32-positive neurons in F6 layer V were not affected by the unilateral M1 lesion.

### Layer V in F3

In contrast to the intact monkeys (no significant IDCD), the IDCD of SMI-32-positive neurons in F3 layer V was significant in all M1 lesioned monkeys except Mk-SL (Fig. 3*C*,*D*). However, the IDCD was not systematically biased toward the same hemisphere (ipsilesional vs contralesional; Fig. 3*D*): two lesioned monkeys (Mk-CE, Mk-RO) exhibited a significantly higher contralesional density of SMI-32 neurons in F3 layer V, corresponding to a negative IDCD, while six injured monkeys (Mk-BI, Mk-DG, Mk-DI, Mk-GE, Mk-JU, Mk-VA) had a significantly higher ipsilesional density of SMI-32 neurons in F3 layer V, corresponding to a positive IDCD. In Mk-SL, the trend of IDCD toward the contralesional side was not statistically significant in F3 layer V (Fig. 3*C*,*D*).

### Layer III in F3 and F6

There was no significant IDCD of SMI-32-positive neurons in layer III of both F6 and F3 (Fig. 3*E*) in intact monkeys. The same was true for F6 layer III in 7 of 9 M1 injured monkeys: only 1 lesioned monkey (Mk-JU) of 9 presented a significantly positive IDCD and the histological analysis was not performed in another monkey (Mk-SL) because of missing histological sections.

There was a statistically significant negative IDCD of SMI-32-positive neurons in F3 layer III in two M1 lesioned monkeys (Fig. 3*E*,*F*: Mk-CE, Mk-RO) reminiscent of the negative IDCD found in F3 layer V in these two monkeys (Fig. 3*C*,*D*). In two other monkeys (Mk-GE, Mk-DI), there was a significant positive IDCD in F3 layer III (Fig. 3*E*,*F*), whereas in the other five monkeys, the IDCDs were not statistically significant (Fig. 3*E*,*F*).

Overall, statistically significant IDCD differences were found in 8 of 9 lesioned monkeys in F3 layer V and in 4 of 9 lesioned monkeys in F3 layer III. These results indicate that the unilateral M1 lesion had a larger impact on SMI-32 neurons in F3 layer V (Fig. 3*D*) than in F3 layer III (Fig. 3*F*). A χ^2^ test revealed that the frequency of significant IDCDs in lesioned monkeys is statistically different to the frequency in intact monkeys for F3 layer V IDCDs (*p* = 0.002, χ^2^ = 9.244, df = 1), but not for F3 layer III IDCDs (*p* = 0.109, χ^2^ = 2.568, df = 1).

### Interindividual IDCDs comparison

Interindividual statistical comparisons of IDCDs displayed in Figure 3, *D* and *F*, were performed, using the nonparametric Kruskal–Wallis test (Table 2). In layer V of F3, there were statistically significant differences of IDCDs, especially between monkeys with extended lesion versus ones with small lesion (Table 2; Fig. 3*D*). For example, the animal with the largest cortical lesion (Mk-CE) showed a significant *p* value with all other animals except Mk-SL (Table 2), in line with the IDCDs distribution shown in Figure 3*F*.

### Arborization of layer V basal dendrites

**Figure 4.**
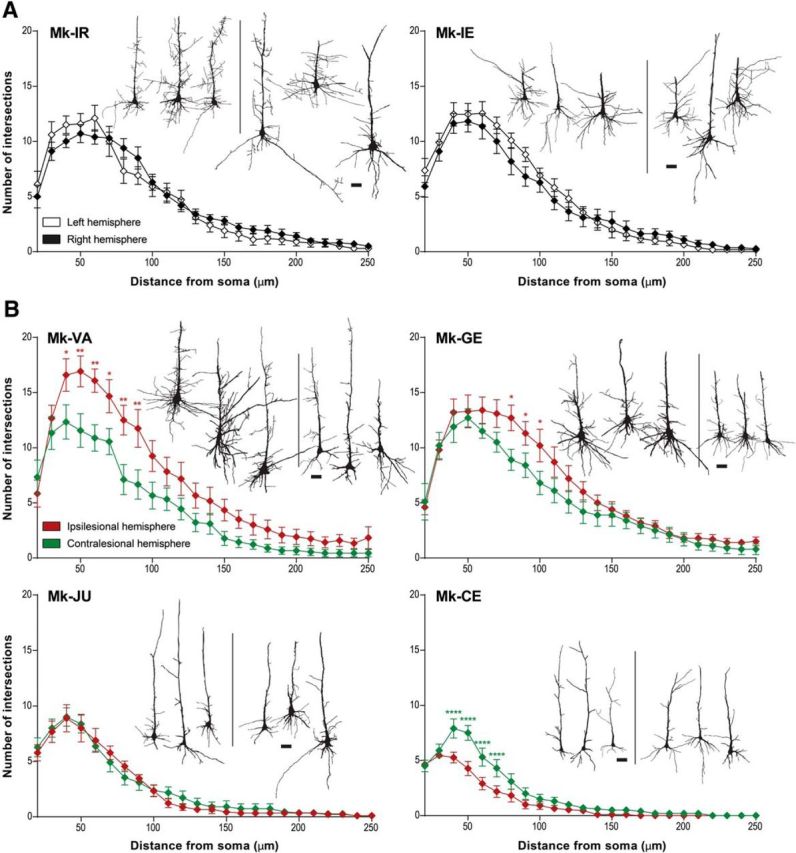
***A***, ***B***, Sholl profiles of basal dendrites of layer V SMI-32-positive neurons in each hemisphere in two intact monkeys (***A***) and in four M1 lesioned monkeys (***B***). For each monkey, histological reconstruction of three analyzed cells from each hemisphere are shown as examples (the 3 cells on the left belong to the left hemisphere and the 3 cells on the right belong to the right hemisphere. All lesioned monkeys presented here had a lesion in the left hemisphere). Scale bars, 20 μm. Intersections were counted at 10 μm intervals from the soma center up to a radius of 250 μm. The curves represent the mean intersection values ± SD. As statistical test, a two-way ANOVA was performed (*p ≤ 0.05, **p ≤ 0.01, ****p ≤ 0.0001).

To assess microstructural changes of the basal dendritic arborization of SMI-32-positive pyramidal neurons located in layer V of F3, a Sholl analysis was performed in two control animals (Mk-IR and Mk-IE) and in four representative lesioned monkeys (Mk-VA, Mk-GE, Mk-JU, and Mk-CE). No interhemispheric difference was observed in control monkeys, with the same increase of dendritic intersection numbers going away from the soma, peaking at a distance of ∼50 μm from the soma, followed by a comparable progressive decrease at larger distances from the soma (Fig. 4*A*). In lesioned animals, interhemispheric differences in the numbers of dendritic intersections were observed in 3 of 4 animals (Fig. 4*B*), with an interhemispheric bias consistent with the IDCD bias already observed for layer V in F3 (Fig. 3*C*,*D*) in these 3 monkeys (Mk-VA, Mk-GE, and Mk-CE). This significant interhemispheric difference of basal dendritic arborization was found only at a distance ranging between ∼40 and 100 μm from the soma (Fig. 4*B*). Even though monkeys Mk-VA and Mk-GE were subjected to an M1 lesion, their dendritic arborization appeared largely as dense as in intact monkeys, whereas in monkeys Mk-CE and Mk-JU, also subjected to a larger M1 lesion, the absolute total numbers of intersections in both hemispheres appeared more sparse.

### Relationship of IDCDs and AUCs in SMI-32-positive neurons with lesion volume and functional recovery

**Figure 5.**
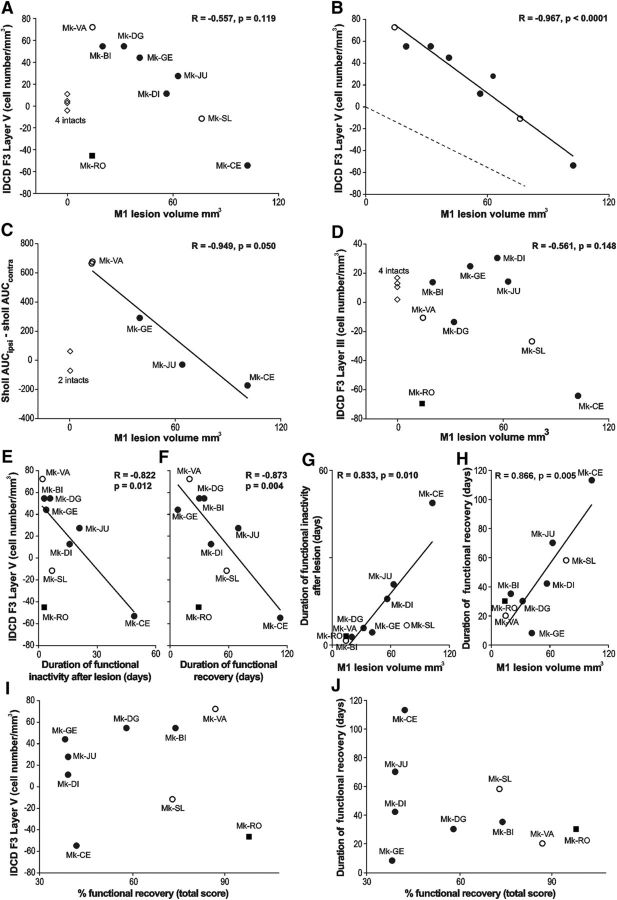
***A***, ***B***, ***D***, The IDCD in F3 (***A***, ***B***, layer V; ***D***, layer III) was plotted as a function of the M1 lesion volume. ***A***, Includes all animals, whereas the intact monkeys, as well as the outlier Mk-RO (square), have been omitted in ***B*** (see Results). ***B***, The dashed line represents the hypothesized interhemispheric cell density asymmetry (see Introduction). ***D***, The regression line and the correlation coefficient do not include the intact monkeys and the outlier Mk-RO. ***C***, The correlation between the dendritic Sholl analysis (AUC) and the M1 lesion volume (without the 2 intact monkeys). ***E***–***H***, Focus on possible behavioral correlates, such as duration in days of functional inactivity after lesion (***E***, ***G***) and duration of functional recovery (***F***, ***H***). These two behavioral parameters were correlated with the IDCD in F3 layer V in ***E*** and ***F***, as well as with the M1 lesion volume in ***G*** and ***H***. ***I***, ***J***, The absence of statistically significant correlation between: the IDCD in F3 layer V and the extent (percentage) of functional recovery (***I***; *r* = 0.049; *p* = 0.900); The duration of functional recovery and the extent of functional recovery (***J***; *r* = 0.355; *p* = 0.349).

The complexity of the basal dendritic arborization was estimated by integrating the number of dendritic intersections at increasing distances from the soma (Fig. 4), yielding an area under the curve (AUC) for each hemisphere. The interhemispheric AUC difference is reported in Figure 5*C*.

To assess whether the morphological changes reported above for the SMI-32 labeled neurons in F3 are related to the properties of the M1 lesion and its consequences, the IDCDs and AUCs were plotted as a function of M1 lesion volume, percentage and duration of functional recovery (Fig. 5*A–D*). The relationship between M1 lesion volumes and IDCDs was clearly different in F3 layer III and in F3 layer V. There was no statistically significant correlation between the median IDCD values in F3 layer III and the M1 lesion volumes (Fig. 5*D*; *r* = −0.561; *p* = 0.148; without taking the outlier Mk-RO into account; changed according to comments. In contrast, IDCDs in F3 layer V were significantly inversely correlated with the M1 lesion volumes when Mk-RO was excluded (Fig. 5*B*; *r* = −0.967; *p* < 0.0001): the smallest M1 lesions were associated with a largely positive IDCD (i.e., ipsilesionally higher SMI-32-positive neuronal density in F3 layer V); progressively larger M1 lesions were accompanied by a decrease of IDCDs, which eventually turned into negative IDCDs for the largest M1 lesions (i.e., contralesionally higher SMI-32-positive neuronal density in F3 layer V). In contrast, in F3 layer III, the same test failed to show any statistically significant difference among all animals (Table 2). As expected, because of the absence of connections between F6 and M1, there was no correlation between the M1 lesion volumes and IDCD median values in F6 layer V (*r* = −0.526, *p* = 0.2252). To distinguish between the two mentioned factors possibly influencing functional recovery, a linear model test including partial correlation showed that both factors have a statistically significant influence on the functional recovery: *p* = 0.0076 for the lesion volume and *p* = 0.035 for the IDCD in F3 layer V.

Mk-RO was not included in these correlation analyses because its M1 lesion procedure was not performed by ibotenic acid infusion at a single time point (a given day), as in all other monkeys, but rather repeated at three time points (days) over several weeks, because the immediate manual dexterity deficit after the first two infusion time points was small and quickly fully reversible. Furthermore, in Mk-RO, the M1 lesion was performed under anesthesia and in presence of anti-epileptic drug phenobarbital (Luminal, 0.2 mg) in contrast to the other monkeys (except Mk-SL who also received post-ibotenic acid infusion phenobarbital following an epileptic seizure). The anti-epileptic treatment in Mk-RO had an unexpectedly large neuroprotective effect against the excitotoxic ibotenic acid in Mk-RO (Snyder et al., 2007). In the other monkeys, the infusion of ibotenic acid was performed in the awake state. Overall, because of the diverging experimental protocol conducted in Mk-RO compared with the other monkeys, the mechanisms of functional recovery, which took place in Mk-RO may thus differ from those observed in the other monkeys subjected to a single-step lesion. These objective experimental differences argue in favor of considering Mk-RO as an outlier (Fig. 5*A*,*B*,*D–J*), whereas results obtained in Mk-SL showed no statistically significant IDCD in F3 layer V, despite a large lesion's volume.

We assessed then the relationship between the duration of functional recovery (Fig. 5*F*), derived from the modified Brinkman board task (total score), and the IDCD median values in F3 layer V. The IDCD median values in F3 layer V were inversely correlated with the duration of functional recovery (Fig. 5*F*; *r* = −0.873; *p* = 0.004). This observation is consistent with the fact that the duration of recovery and the lesion volume are themselves dependent variables (Fig. 5*H*; *r* = 0.866; *p* = 0.005). In contrast, the median IDCD values in F3 layer III were neither correlated with the duration of functional recovery (*p* = 0.06, without Mk-RO), nor with the M1 lesion volumes (Fig. 5*D*).

Although the IDCD median values in F3 layer V were inversely correlated with both the M1 lesion volume (Fig. 5*B*) and the duration of functional recovery (Fig. 5*F*), the IDCD median values in F3 layer V were not significantly correlated with the percentage of functional recovery (defined in Fig. 1*F*) assessed with the total score (*p* = 0.73; Fig. 5*I*), in the modified Brinkman board task (also excluding Mk-RO here). The animals presenting a large M1 lesion (such as Mk-CE) recovered to a lesser extent the precision grip ability than monkeys subjected to a smaller lesion (Wyss et al., 2013). In addition, monkeys with a large M1 lesion exhibited postlesion a change of manual dexterity strategy to palliate the strong movement deficit of the thumb finger compared with animals with smaller lesion (such as Mk-VA), which recovered their original opposition of thumb and index finger. In contrast, after large M1 lesion (e.g., Mk-CE), the function of the thumb was less recovered, preventing recovery of the original opposition of thumb and index finger; instead, the successful retrieval postlesion was more dependent on the index finger, opposing either a largely passive thumb or other parts of the hand (e.g., palm). Another outcome was that after large M1 lesion, the monkey was clearly more proficient in the retrieval of pellets from vertical slots rather than horizontal slots in the modified Brinkman board task.

In the present model of M1 lesion restricted to the hand area, there was no correlation between the percentage of functional recovery in the modified Brinkman board task and the duration of functional recovery (Fig. 5*J*). In other words, a longer duration of functional recovery did not mean a better functional recovery. Finally, the IDCD median values were not correlated with the time interval in days (Table 1) between the M1 lesion and the day of euthanasia, in other words the survival time postlesion.

## Discussion

Microscopic examination of pyramidal layers in the SMA (F3 and F6) in macaque monkeys subjected to a unilateral lesion of the hand representation in M1 showed a significant interhemispheric asymmetry of SMI-32 staining density in layer V of F3 in 8 of 9 monkeys. In F3 layer III such an interhemispheric asymmetry was observed in only four monkeys (with a comparable interhemispheric bias as in layer V). The interhemispheric asymmetries of SMI-32 staining density were consistent with data derived from basal dendritic arborization complexity analyses conducted in a subgroup of four lesioned monkeys. In contrast, asymmetry was observed neither in F6 layer V in M1 lesioned animals, nor in F3 and F6 in intact animals. Interestingly, the extent and direction of the interhemispheric asymmetries of SMI-32 labeled cells in F3 layer V, but not in F3 layer III, was correlated with the lesion volume in M1, and with the duration of the functional recovery of manual dexterity.

### Our four working hypotheses were largely verified by the data

(1) A unilateral lesion of M1 indeed impacted at distance onto SMA. This effect was different on both the ipsilesional and contralesional hemispheres, with an interhemispheric asymmetry of density of SMI-32 labeled neurons in F3 layer V (Figs. 2, 3). (2) The effect of the M1 lesion on SMA on both sides (in F3 layer V) was correlated with the lesion volume (Fig. 5*B*). (3) Consistent with the role of SMA in the functional recovery, the impact of the M1 lesion on both SMAs was correlated with the volume of the M1 lesion and with the duration of functional recovery. (4) The impact of the unilateral M1 lesion was different onto F3 (SMA-proper) and F6 (pre-SMA). Indeed, the M1 lesion impacted at distance on F3 but not on F6, in line with their connectional properties with M1 (Figs. 1*A*, 2, 3, 5).

### Diaschisis?

The present observation is reminiscent of the concept of diaschisis (Von Monakow, 1914), defined as a “loss of function and electrical activity in an area of the brain because of a lesion in a remote area that is neuronally connected with it”. The unilateral lesion of M1 hand area may indeed have affected the function of neurons in SMA (mainly F3), differently in the ipsilateral and contralateral hemispheres, resulting in differently modified phenotypes (morphological characteristics) between both hemispheres. Such differential interhemispheric change of phenotype may have modified the affinity of F3 layer V neurons for the SMI-32 antibody, resulting in the observed interhemispheric asymmetry of density of the SMI-32-positive neurons. Von Monakow (1914) actually distinguished between an ipsilateral diaschisis and a commissural diaschisis. The interpretation of a modification of SMI-32 antibody affinity is consistent with the observation that the absolute numbers of pyramidal neurons in layers III and V in the M1 lesioned monkeys bilaterally was in the same range as in intact monkeys (Fig. 3), suggesting an absence of loss of neurons in F3 as a result of the M1 lesion. The interpretation of a change of phenotype of F3 layer V neurons, leading to an interhemispheric asymmetry of SMI-32 antibody affinity, is supported by the basal dendritic arborization data. The directions of interhemispheric differences in dendritic arborization complexity (Fig. 4) were consistent with the direction of the IDCD.

### Correlation between IDCD and M1 lesion, respectively duration of functional recovery

Shortly after lesion in M1 hand area, the ibotenic acid is expected to destroy neurons in M1 but preserve the axons in the lesion territory (Coffey et al., 1988). As a result, SMA should be impacted by decreased inputs from M1. Moreover, a possible lesion signal coming from M1 may be transported retrogradely along the axons projecting from SMA to M1 because these axons have been partly deprived of their target in M1. Due to denser anatomical interconnections between M1 and the ipsilateral SMA compared with the contralateral SMA in intact macaque monkeys, the effects of a unilateral M1 lesion should be different across both hemispheres, and should depend on the lesion volume in M1. A purely connectional model would predict that a tiny M1 lesion has hardly no effect on SMA neuronal density, whereas larger lesion volumes induce a progressively increasing interhemispheric asymmetry in SMI-32-positive neurons, yielding more and more negative IDCDs as shown in Figure 5*B* (dashed line, arbitrary slope), and increased trophic loss of connected neurons in M1.

The actual data (Fig. 5*B*, solid regression line; *r* = −0.967) show indeed a negative correlation between the IDCD and the lesion volume, but the regression line is shifted toward positive IDCDs, meaning that there is a strong asymmetry biased toward the ipsilesional side, and this bias is the most extreme in case of small lesions (not considering the outlier Mk-RO). Obviously, the actual data cannot be only explained by the above-mentioned purely connectional model. Other parameters may play a role: first, some diaschisis, corresponding to a functional effect of the lesion at distant but directly connected areas, may have resulted in a change of the phenotype of pyramidal neurons in F3. Second, the histological data refer to the brain state several months after the lesion, when postlesion plasticity has taken place, allowing some functional recovery. Postlesion plasticity may have contributed to modify the phenotype of the neurons in F3, making it possible for SMA to play a role in the functional recovery (McNeal et al., 2010; Morecraft et al., 2015), in accordance with the observation that IDCD is inversely correlated with the duration of functional recovery (Fig. 5*F*).

The correlation between IDCD asymmetry and the duration of functional recovery is also reminiscent of the controversy related to the respective contributions of the ipsilesional versus contralesional hemispheres in the functional recovery following a unilateral lesion of M1 (Netz et al., 1997; Liu and Rouiller, 1999; Carey et al., 2002; Johansen-Berg et al., 2002; Ward et al., 2003; Luke et al., 2004; Ward and Cohen, 2004; Biernaskie et al., 2005; Lotze et al., 2006; Bradnam et al., 2012, 2013; Young et al., 2014; Touvykine et al., 2016; for review, see Auriat et al., 2015; Dancause et al., 2015). There is evidence that the contralesional hemisphere is playing a more important role in the early phase of functional recovery (Rehme et al., 2011; Dancause et al., 2015; Savidan et al., 2017).

In macaque monkeys, the crucial role played by the extent of the lesion and its position in the motor cortex in the functional recovery has been reported earlier (Darling et al., 2009, 2011; Kaeser et al., 2010, 2011; Wyss et al., 2013; Morecraft et al., 2015, 2016). Our results show here that a small M1 cortical lesion was associated with a higher density of SMI-32-positive neurons in ipsilesional SMA, increasing then the M1 lesion size resulted in reducing this interhemispheric asymmetry up to reaching an inversion of this asymmetry, in favor of the contralesional hemisphere, with a very large M1 lesion (Fig. 5*B*). We also observed a strong negative correlation (*r* = −0.949; Fig. 5*C*) between the volume of the M1 lesion and the complexity of the basal dendritic arborization. The extent of the cortical lesion is therefore considered as a major factor affecting the neuronal density of SMI-32-positive neurons and their morphology in SMA.

### Difference in interhemispheric asymmetry between layer III and layer V in F3

Cortical lesion targeting M1 hand representation led to morphological changes occurring mainly in F3 layer V in the form of interhemispheric asymmetry of neuronal density assessed with SMI-32 (in 8 of 9 monkeys, the trend being statistically nonsignificant in 1 animal, but still in the same direction as in the other 8 animals), paralleled with less systematic changes in F3 layer III in the same direction as in F3 layer V (in 4 of 9 monkeys). In contrast to F3 layer V, the IDCD in F3 layer III was not correlated to the M1 lesion volume. Both layers are the source and target of corticocortical projections. In addition, some pyramidal neurons located in layer V, but not in layer III, do project to the spinal cord (Dum and Strick, 1996; Rouiller et al., 1996; Maier et al., 2002). Interestingly, the CS projection from the ipsilesional F3 is subjected to axonal sprouting in the cervical cord after unilateral M1 lesion (McNeal et al., 2010; Morecraft et al., 2015), possibly underlying the incomplete functional recovery. This anatomical property may explain the stronger interhemispheric density asymmetry observed in F3 layer V compared with F3 layer III. Indeed, we hypothesize that the larger the M1 lesion, the more M1 CS axon terminals in the cervical cord degenerate, leaving more space for CS axons originating first from the ipsilesional F3, layer V, to sprout and extend in the cervical cord after the M1 lesion. This mechanism is expected to be proportional to the lesion volume, as is the change of phenotype in F3 layer V pyramidal neurons.

## Conclusion

SMI-32, the marker of pyramidal neurons in layers III and V in motor cortical areas, does not exhibit a stable level of immunoreactivity in SMA across both hemispheres, but rather an interhemispheric asymmetry that is more pronounced in layer V than layer III, following a unilateral lesion of the M1 hand area. Such asymmetry may reflect neuronal plastic changes possibly underlying functional recovery following such a lesion. As such, SMI-32 labeling represents a promising tool to tentatively identify remote intact cortical areas contributing to the functional recovery from the lesion. As the PMv-r (F5) has been shown to be also involved in the functional recovery from M1 lesion (Liu and Rouiller, 1999; Dancause et al., 2005; Hoogewoud et al., 2013), one may expect to see there a similar interhemispheric imbalance of SMI-32 labeling, may be also related to the volume of the lesion, although this may be challenged by the specificity of the CS projection from F5 to terminate higher in the cervical cord (C3–C4) than the most relevant segments for hand muscles' control (C7–T2).
